# Exploring RNA modifications, editing, and splicing changes in hyperuricemia and gout

**DOI:** 10.3389/fmed.2022.889464

**Published:** 2022-09-06

**Authors:** Chung-Ming Huang, Yu-Chia Chen, I-Lu Lai, Hong-Da Chen, Po-Hao Huang, Siang-Jyun Tu, Ya-Ting Lee, Ju-Chen Yen, Chia-Li Lin, Ting-Yuan Liu, Jan-Gowth Chang

**Affiliations:** ^1^Center for Precision Medicine, China Medical University Hospital, Taichung, Taiwan; ^2^Division of Immunology and Rheumatology, Department of Internal Medicine, China Medical University Hospital, Taichung, Taiwan; ^3^Graduate Institute of Integrated Medicine, College of Chinese Medicine, China Medical University, Taichung, Taiwan; ^4^Epigenome Research Center, China Medical University Hospital, Taichung, Taiwan; ^5^Million-Person Precision Medicine Initiative, Department of Medical Research, China Medical University Hospital, Taichung, Taiwan

**Keywords:** gout, RNA modifications, RNA splicing, RNA editing, epigenomic regulations

## Abstract

Hyperuricemia and gout are two of the most common metabolic disorders worldwide; their incidence is increasing with changes in lifestyle, and they are correlated with many diseases, including renal and cardiovascular diseases. The majority of studies on hyperuricemia and gout have focused on the discovery of the associated genes and their functions and on the roles of monocytes and neutrophils in the development of gout. Virtually no studies investigating the epigenomics of gout disease or exploring the clinical significance of such research have been conducted. In this study, we observed that the expression of enzymes involved in RNA modifications or RNA editing was affected in uric acid (UA)- or monosodium urate (MSU)-treated cell lines. RNA alternative splicing and splicing factors were also affected by UA or MSU treatment. We used transcriptome sequencing to analyze genome-wide RNA splicing and RNA editing and found significant changes in RNA splicing and RNA editing in MSU- or UA-treated THP-1 and HEK293 cells. We further found significant changes of RNA modifications, editing, and splicing in patients with gout. The data indicate that RNA modifications, editing, and splicing play roles in gout. The findings of this study may help to understand the mechanism of RNA splicing and modifications in gout, facilitating the development of new diagnostic and therapeutic strategies.

## Introduction

Hyperuricemia and gout are two of the most common metabolic diseases worldwide. Gout is more common in men than in women, and the male to female ratio ranges from 3:1 to 10:1. The global prevalence and incidence of gout are in the ranges of 1–4% and 0.1–0.3%, respectively (1). In Taiwan, the prevalence and incidence of gout are 6.24% and 2.74 per 1,000 person-years, respectively. The overall prevalence of gout is 2.9-fold more in men than in women (2). The prevalence and incidence of gout are increasing with the increasing richness of diets in recent years and other lifestyle factors.

Gout is a chronic disease characterized by the deposition of monosodium urate (MSU) crystals in joints and soft tissues, and its cause is related to an imbalance between urate intake/production and excretion, which leads to urate accumulation and crystallization in tissues (3). MSU crystals trigger the activation of the NLRP3 inflammasome and the release of proinflammatory cytokines such as interleukin (IL)-1β, tumor necrosis factor (TNF)-α, IL-8, IL-17, IL-10, and IL-37. IL-1β is the critical inflammatory mediator induced by MSU crystals (4–7).

Although the mechanism by which inflammation is activated in gout has been discovered, the mechanism of epigenomic regulation of gout remains unclear. Technological breakthroughs have led to epigenomics becoming one of the most rapidly expanding fields in biology (8). Epigenetic regulations include miRNA, histone modifications, RNA editing, RNA modifications, and RNA splicing. The role of abnormal epigenomic regulation in the pathogenesis of various diseases, including cancers, autoimmune diseases, and neurological diseases, has been widely reported (9, 10). However, studies on epigenomic mechanisms in gout and hyperuricemia are scarce, and only several studies on microRNA and DNA methylation genes such as miR-155 and miR-146a have been reported (11–18). Other epigenomic regulations may also play important roles in hyperuricemia and gout. Through this study, we aimed to investigate the epigenomic changes in hyperuricemia and gout.

## Materials and methods

### Cell culture and treatment

THP-1 cells were grown in lipopolysaccharide-free complete RPMI medium containing 10% fetal bovine serum. HEK293 cells were grown in Dulbecco’s modified Eagle’s medium containing 10% fetal bovine serum. HUVEC cells were grown in Cascade Biologics Medium 200 (Gibco, Thermo Fisher Scientific, Waltham, MA, United States) plus Cascade Biologics Low Serum Growth Supplement (Gibco, Thermo Fisher Scientific, Waltham, MA, United States) on a gelatin-coated dish. Cells were grown at 37°C in an incubator with 5% CO_2_. Uric acid (UA) was dissolved in 1 N sodium hydroxide and diluted in culture media to 3.5, 7, and 10.5 mg/dL. Media containing UA were adjusted to pH 7.4. MSU was dissolved in phosphate-buffered saline and diluted in culture media to 3.5, 7, and 10.5 mg/dL. Cells were seeded on a culture dish and treated with UA- or MSU-containing media for 48 h.

### RNA extraction and reverse transcription and real-time polymerase chain reaction

RNA was extracted from UA- or MSU-treated cells or from the buffy coat of patients with gout by using TRIzol Reagent (Thermo Fisher Scientific, Waltham, MA, United States) or the NucleoSpin RNA kit (Macherey-Nagel, Dueren, Germany), according to the manufacturer’s instructions. Two micrograms of RNA were subjected to reverse transcription polymerase chain reaction (RT-PCR) using the Applied Biosystems High-Capacity cDNA Reverse Transcription Kit (Thermo Fisher Scientific, Waltham, MA, United States), according to the manufacturer’s instructions. Real-time polymerase chain reaction (PCR) was performed on the Roche LightCycler 480 Real-Time PCR System using Universal ProbeLibrary System (Roche, Basel, Switzerland). The PCR process involved the following steps: initial denaturation at 94°C for 5 min, followed by 35 cycles of 94°C for 30 s, 60°C for 45 s, 72°C for 1 min, and final extension at 72°C for 7 min. The PCR products were separated by 3% agarose gel electrophoresis. The intensity of the PCR products was analyzed using LabWorks Image Acquisition and Analysis Software (UVP BioImaging Systems, Upland, CA, United States). The primers for RT-PCR and real-time PCR are listed in Supplementary Table 1.

### Statistical analysis

Statistical analysis was performed using Microsoft Excel 2010 (Microsoft, Redmond, WA, United States) and GraphPad Prism 5.01 (GraphPad Software, San Diego, CA, United States). Student’s *t*-tests were conducted to evaluate differences between groups. A probability of less than 0.05 was considered to be statistically significant. *P < 0.05, **P < 0.01, and ***P < 0.0001.

### Protein extraction and western blotting

Proteins were extracted from UA- or MSU-treated cells or from the buffy coat of patients with gout by using RIPA lysis buffer (50 mM Tris, pH 8.0, 150 mM NaCl, 1 mM EDTA, 1% NP40, 1% sodium deoxycholate, 0.1% SDS) and were analyzed through SDS-PAGE: the proteins were transferred onto polyvinylidene difluoride (PVDF) membranes (Bio-Rad, Hercules, CA, United States) after electrophoresis. The PVDF membranes were blocked with 5% bovine serum albumin (BSA) solution in *Tris*-buffered saline with Tween 20 (TBST) for 60 min. The PVDF membranes were incubated with the primary antibody in 1% BSA overnight and then incubated with horseradish peroxidase–conjugated secondary antibody for 60 min. After washing three times with TBST, the PVDF membranes were exposed to the ECL Plus substrate (GE Healthcare Amersham, Fisher Scientific, Göteborg, Sweden) for 5 min. Chemiluminescence signals were detected, and the expression levels were analyzed using LabWorks Image Analysis Software. The primary antibodies used were anti-PUS1 (Invitrogen, Thermo Fisher Scientific, Waltham, MA, United States), anti-PUS7 (Invitrogen, Thermo Fisher Scientific, Waltham, MA, United States), anti-NSUN3 (Abcam, Cambridge, United Kingdom), anti-NSUN4 (Abcam, Cambridge, United Kingdom), anti-METTL14 (Abcam, Cambridge, United Kingdom), anti-YTHDC1 (Abcam, Cambridge, United Kingdom), anti-YTHDF3 (Abcam, Cambridge, United Kingdom), anti-ADAR1 (Abcam, Cambridge, United Kingdom), anti-RED1 (also known as ADAR2, Abcam), anti-SRPK1 (BD Biosciences, Franklin Lakes, NJ, United States), anti-SRPK2 (BD Biosciences, Franklin Lakes, NJ, United States), anti-SF2/ASF (Zymed Laboratories, South San Francisco, CA, United States), anti-hnRNPA1 (Sigma-Aldrich), anti-Sam68 (Santa Cruz, Dallas, TX, United States), anti-histone H1 (GeneTex, Irvine, CA, United States), anti-ß-actin (GeneTex, Irvine, CA, United States), and anti-tubulin (GeneTex, Irvine, CA, United States).

### Samples of patients with gout

Blood samples obtained from 67 patients with gout were approved by the China Medical University and Hospital Research Ethics Committee (CMUH108-REC2-051). Approximately 10 mL of blood was collected in an EDTA tube, and buffy coat was used for RNA and protein extraction.

### Transcriptome sequencing

That RNA was of high quality (RNA integrity number, RIN > 8) was confirmed using the Agilent Bioanalyzer 4200 (Agilent Technologies, Santa Clara, CA, United States). One microgram of RNA was used for library preparation of the TruSeq Stranded mRNA Library Prep kit (Illumina, San Diego, CA, United States), according to the manufacturer’s instructions. Briefly, mRNA was purified using poly-A magnetic beads and fragmented through enzyme treatment. Subsequently, double-strand cDNA synthesis, end-repair, adaptor ligation, and enrichment PCR were performed. Samples were subjected to 2 × 150-bp paired-end sequencing using the Illumina NovaSeq 6000 platform.

### Bioinformatics analysis

Base calling and quality scoring were performed with an updated implementation of Real-Time Analysis on the NovaSeq 6000 system. We used bcl2fastq Conversion Software (v2.20.0.422) to demultiplex data and convert BCL files to FASTQ files. To facilitate downstream analysis, a quality control (QC) step was performed that involved the use of Trimmomatic tools (v0.39) (19). Sequencing primers and adapters in the reads were trimmed off, and a read was dropped out if the average quality score was less than 20 or the length of the read was shorter than 100 bp. Transcriptome sequencing reads were aligned to human genome hg38 with reference annotation GENCODE version 30 by using STAR (v2.7.5a) (20). featureCounts (v35) (21) and edgeR (v 3.32.1) (22) were used to quantify and normalize the mRNA expression level. To identify alternative splicing events (ASEs), rMATS (v4.1.0) (23) was applied to alignment results from STAR. A total of five types of ASEs were observed, namely annotated, skipped exon (SE), mutually exclusive exons (MXE), alternative 3′ splice site (A3SS), alternative 5′ splice site (A5SS), and retained intron (RI). To analyze RNA editing, whole genome sequencing (WGS) data were obtained from NCBI SRA (HEK293: SRR2123657, THP-1: SRR8670675); fastp (v0.20.1) (24) was used for QC. WGS reads were aligned to human genome hg38 with reference annotation GENCODE version 30 by using BWA-MEN (v0.7.17) (25) and were then sorted to generate an index by using SAMtools (v 1.9) (26). RedITools (v 1.0) (27) plus the relevant protocol (28) was used for RNA editing. QC was performed by filtering out the editing events with read counts less than 30 and editing ratios less than 0.1.

## Results

### Effect of uric acid and monosodium urate on RNA modifications

**FIGURE 1:**
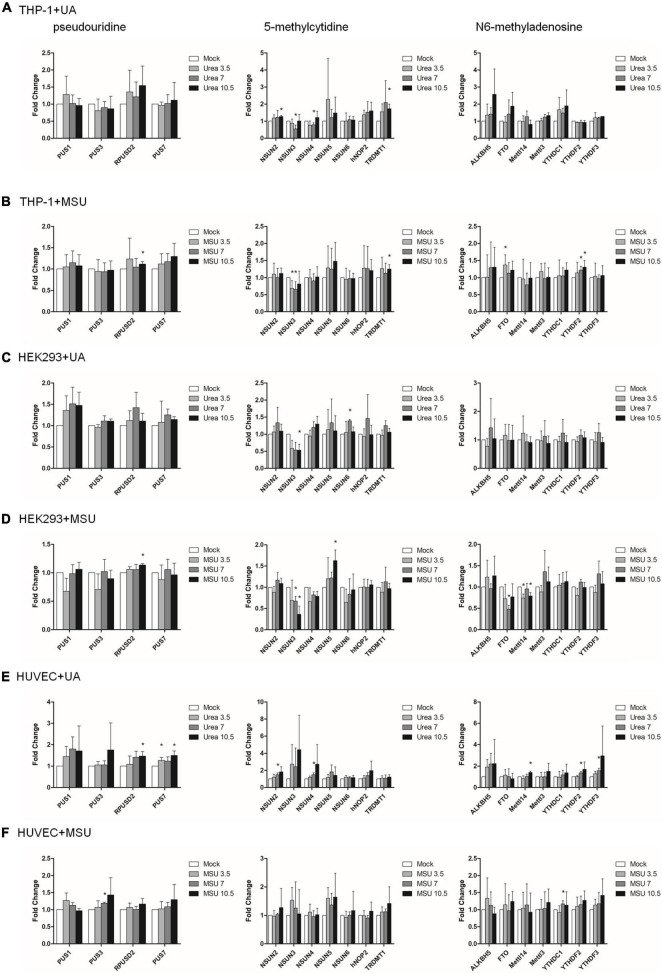
RNA expressions of RNA modification enzymes were affected in MSU- or UA-treated cell lines. The expressions of enzymes involved in pseudouridine (left), 5-methylcytidine (middle), and N6-methyladenosine (right) in UA-treated THP-1 cells **(A)**, MSU-treated THP-1 cells **(B)**, UA-treated HEK293 cells **(C)**, MSU-treated HEK293 cells **(D)**, UA-treated HUVEC cells **(E)**, and MSU-treated HUVEC cells **(F)** were quantitated through real-time PCR. Error bars represent standard deviation. The results of three independent experiments were averaged to obtain the final results. *P < 0.05 by Student’s *t*-test, compared with mock cells.

**FIGURE 2:**
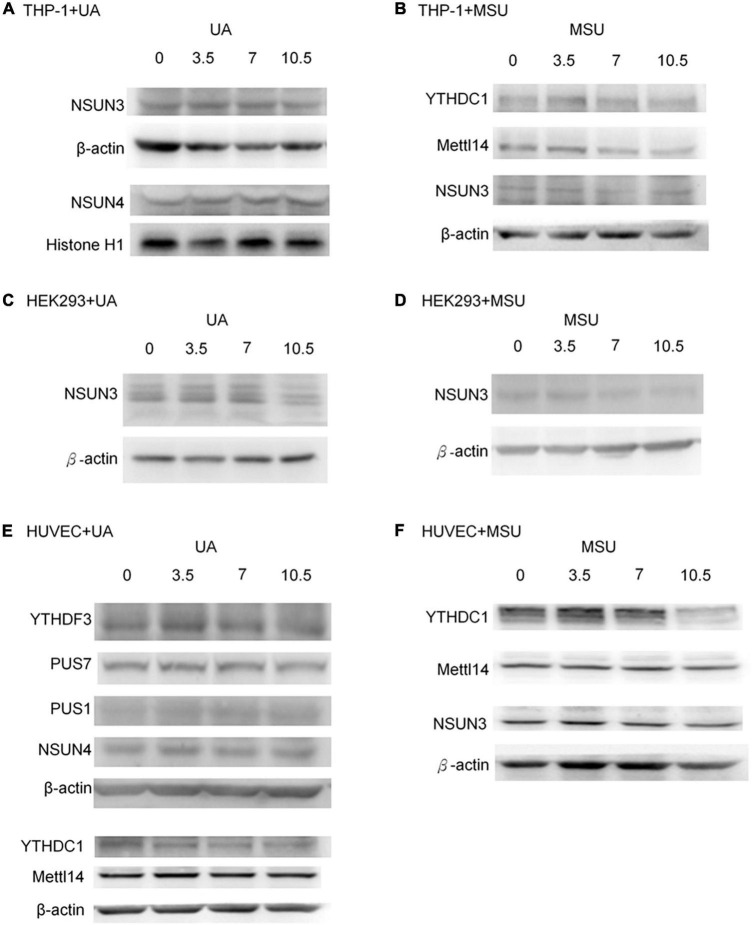
Protein expressions of RNA modification enzymes were affected in MSU- or UA-treated cell lines. The expressions of RNA modification enzymes that changed with RNA levels were detected through Western blotting. **(A)** NSUN3 and NSUN4 were detected in UA-treated THP-1 cells. **(B)** YTHDC1, METTL14, and NSUN3 were detected in MSU-treated THP-1 cells. **(C,D)** NSUN3 was detected in UA-and MSU-treated HEK293 cells. **(E)** YTHDF3, PUS7, PUS1, NSUN4, YTHDC1, and METTL14 were detected in UA-treated HUVEC cells. **(F)** YTHDC1, METTL14, and NSUN3 were detected in MSU-treated HUVEC cells. β-actin and histone H1 served as the internal control. Representative results from three independent experiments are presented.

Epigenomic regulations include DNA and RNA levels, and over 17 and 160 types of modifications have been detected in DNA and RNA, respectively (8, 29, 30). Previous studies have shown that RNA modifications modulate innate immune response and are linked to many human diseases (31, 32). We hypothesized that RNA modifications may be regulated in hyperuricemia and gout. To identify the effect of UA and MSU on RNA modifications, we treated the THP-1 monocyte cell line, HEK293 embryonic kidney cell line, and HUVEC umbilical vein epithelial cell line with UA or MSU for 48 h. These cell lines were selected because the evidence indicates that hyperuricemia and gout are involved in the development of chronic kidney disease and hypertension. In addition, MSU crystal-stimulated monocytes and macrophages secret IL-1 and initiate gouty inflammation. Subsequently, we measured the expression levels of enzymes involved in RNA modifications, including pseudouridine, 5-methylcytosine, and N6-methyladenosine; these enzymes have been associated with human disease-related RNA modifications. We determined that UA and MSU had diverse effects on RNA modifications in different cell lines (Figure 1). The expression of enzymes involved in 5-methylcytosine was affected in THP-1 cells treated with UA (Figure 1A). The effect of MSU on RNA modifications in THP-1 cells was ubiquitous in pseudouridine, 5-methylcytosine, and N6-methyladenosine (Figure 1B). The expressions of the enzymes involved in 5-methylcytosine in UA-treated HEK293 cells changed significantly (Figure 1C). The effect of MSU on RNA modifications in HEK293 cells was ubiquitous in pseudouridine, 5-methylcytosine, and N6-methyladenosine (Figure 1D). UA treatment of HUVEC cells altered the expressions of the enzymes involved in pseudouridine, 5-methylcytosine, and N6-methyladenosine (Figure 1E). MSU treatment of HUVEC cells affected the expressions of the enzymes involved in pseudouridine and N6-methyladenosine (Figure 1F). The results indicated that both UA and MSU affected RNA modifications. We further detected the protein expression levels of RNA modification enzymes that were affected by RNA levels. We found no significant changes of NSUN3 and NSUN4 protein expressions in THP-1 cells treated with UA (Figure 2A). The expression of METTL14 decreased in THP-1 cells treated with MSU; however, no changes were observed in the expressions of YTHDC1 and NSUN3 (Figure 2B). The expression of NSUN3 decreased in HEK293 cells treated with UA or MSU (Figures 2C,D). No significant changes in protein expressions of RNA modification enzymes were observed in HUVEC cells treated with UA or MSU (Figures 2E,F). The quantitative results are presented in Supplementary Figure 1.

### Effect of uric acid and monosodium urate on RNA editing

**FIGURE 3:**
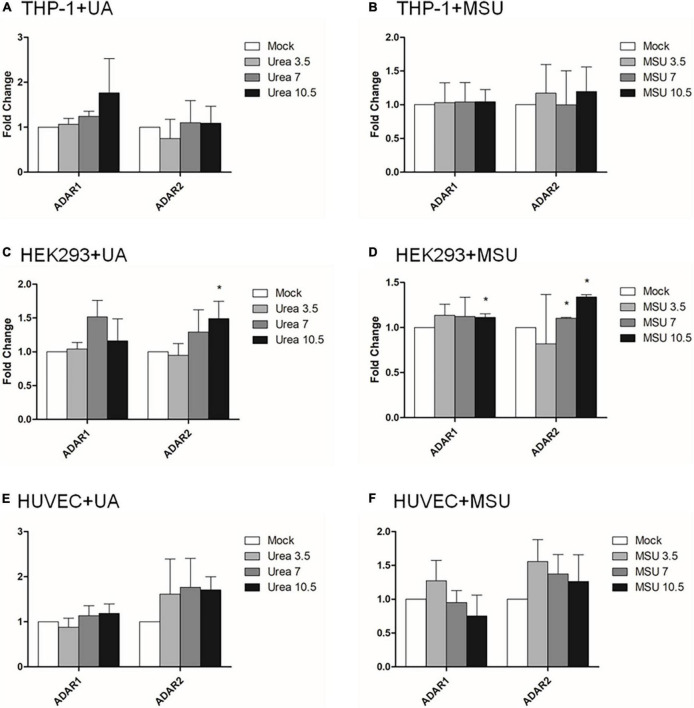
RNA expressions of RNA editing enzymes were affected in MSU- or UA-treated HEK293 cells. The expressions of *ADAR1* and *ADAR2* in UA-treated THP-1 cells **(A)**, MSU-treated THP-1 cells **(B)**, UA-treated HEK293 cells **(C)**, MSU-treated HEK293 cells **(D)**, UA-treated HUVEC cells **(E)**, and MSU-treated HUVEC cells **(F)** were quantitated through real-time PCR. Error bars represent standard deviation. The results of three independent experiments were averaged to obtain the final results. *P < 0.05 by Student’s *t*-test, compared with mock cells.

**FIGURE 4:**
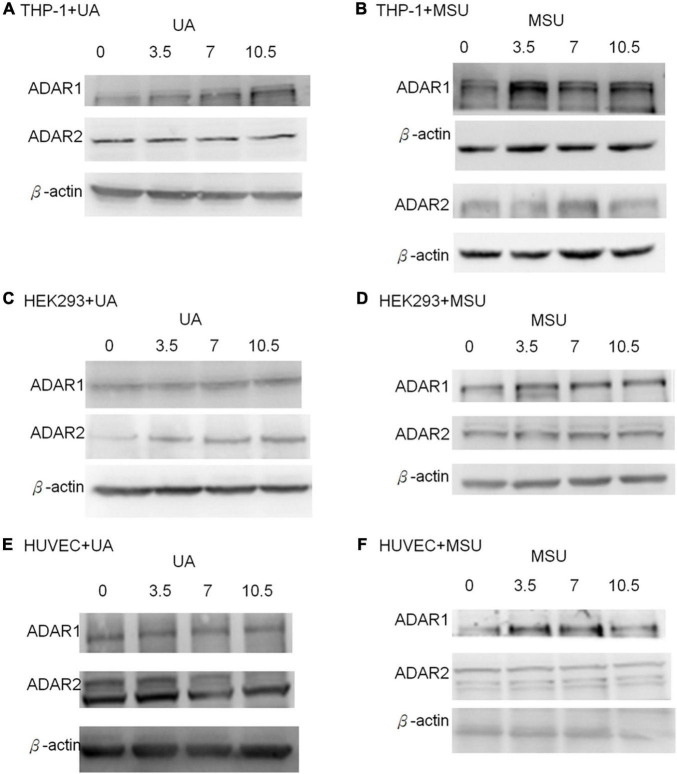
Protein expressions of RNA editing enzymes were affected in MSU- or UA-treated cell lines. The expressions of ADAR1 and ADAR2 were detected through Western blotting in UA-treated THP-1 cells **(A)**, MSU-treated THP-1 cells **(B)**, UA-treated HEK293 cells **(C)**, MSU-treated HEK293 cells **(D)**, UA-treated HUVEC cells **(E)**, and MSU-treated HUVEC cells **(F)**. β-actin served as the internal control. Representative results from three independent experiments are presented.

**FIGURE 5:**
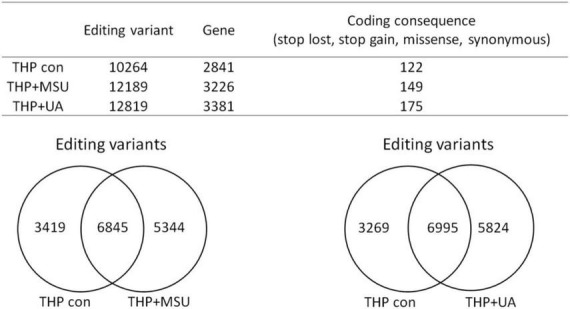
Genome-wide analysis of RNA editing in MSU- or UA-treated THP-1 cells. The upper panel shows the numbers of variants and genes with RNA editing in mock, MSU-treated, or US-treated THP-1 cells. The numbers of variants in the coding sequence were further analyzed. RNA editing affected the coding sequence, including gain of stop codon, loss of stop codon, missense mutations, and non-synonymous mutations. The left lower panel displays the common variants and unique variants in untreated and MSU-treated THP-1 cells, and the right lower panel presents the common variants and unique variants in untreated and UA-treated THP-1 cells.

**FIGURE 6:**
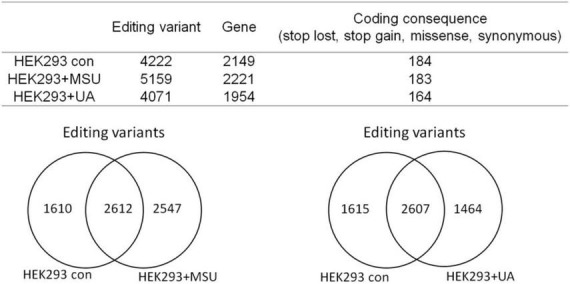
Genome-wide analysis of RNA editing in MSU- or UA-treated HEK293 cells The upper panel displays the numbers of variants and genes with RNA editing in mock, MSU-treated, or US-treated HEK293 cells. The numbers of variants in a coding sequence were further analyzed. RNA editing affected the coding sequence, including gain of stop codon, loss of stop codon, missense mutations, and non-synonymous mutations. The left lower panel presents the common variants and unique variants in untreated and MSU-treated HEK293 cells, and the right lower panel displays the common variants and unique variants in untreated and UA-treated HEK293 cells.

RNA editing is another critical mechanism in gene regulation. RNA editing is different from other RNA modifications because it alters the cellular fate of RNA molecules and their sequence relative to the genome. Adenosine to inosine (A-to-I) RNA editing is the most common type of RNA editing in vertebrates (higher eukaryotes). This editing reaction is catalyzed by the adenosine deaminase acting on RNA (ADAR) protein family (33). A-to-I RNA editing has been determined to be involved in many biological functions; one of them is innate immune response (34–36). According to these studies, A-to-I RNA editing may be regulated in hyperuricemia and gout. To identify the effect of UA and MSU on A-to-I RNA editing, we detected the expression levels of enzymes involved in A-to-I RNA editing, including ADAR1 and ADAR2, and found no significant changes of *ADAR1* and *ADAR2* expressions in THP-1 and HUVEC cells treated with UA or MSU (Figures 3A,B,E,F). The expression of *ADAR2* increased in HEK293 cells treated with UA (Figure 3C), and the expressions of *ADAR1* and *ADAR2* increased in HEK293 cells treated with MSU (Figure 3D). We also detected the protein expressions of ADAR1 and ADAR2 in three cell lines treated with UA or MSU and observed that the expression of ADAR1 increased in THP-1 cells treated with UA and MSU, but no change was observed in the expression of ADAR2 (Figures 4A,B). Increases in the expression of ADAR2 in HEK293 cells treated with UA were observed (Figure 4C), whereas no changes of ADAR1 and ADAR2 in HEK293 cells treated with MSU were observed (Figure 4D). No changes in the expressions of ADAR1 and ADAR2 were observed in HUVEC cells treated with UA (Figure 4E), but the expression of ADAR1 increased in HUVEC cells treated with MSU (Figure 4F). The quantitative results are presented in Supplementary Figure 2. These findings indicate that not only RNA modifications but also RNA editing was affected by UA or MSU treatment. To further explore RNA editing, we analyzed genome-wide RNA editing by transcriptome sequencing in UA- or MSU-treated THP-1 and HEK293 cells. We found 10,264 editing variants in THP-1 cells and 12,189 and 12,819 editing variants in MSU-treated THP-1 cells and UA-treated THP-1 cells, respectively (Figure 5); 6,845 editing variants were commonly observed in untreated THP-1 and MSU-treated THP-1 cells, and 3,419 and 5,344 unique editing variants were observed in untreated THP-1 and MSU-treated THP-1 cells, respectively. A total of 6,995 common variants and 3,269 and 5,824 unique variants were observed in untreated THP-1 and UA-treated THP-1 cells, respectively (Figure 5). A total of 4,222, 5,159, and 4,071 editing variants were observed in untreated, MSU-treated, and UA-treated HEK293 cells, respectively (Figure 6); 2,612 editing variants were common in untreated and MSU-treated HEK293 cells, and 1,610 and 2,547 unique editing variants were observed in untreated THP-1 and MSU-treated HEK293 cells, respectively. In total, 2,607 common variants and 1,615 and 1,464 unique variants were observed in untreated HEK293 and UA-treated HEK293 cells, respectively (Figure 6). Subsequently, we analyzed editing variants correlated with gene expression levels. Four genes exhibited expression level changes with RNA editing variants in MSU-treated THP-1 cells, namely *GATD3B*, *GLO1*, *IL21R*, and *SIGLEC6*. In UA-treated THP-1 cells, four genes exhibited changed expression levels with RNA editing variants, namely *FADS2*, *IL21R*, *SIGLEC11*, and *SIGLEC6*. In total, 78 and four genes exhibited changed expression levels in MSU- and UA-treated HEK293 cells, respectively (Supplementary Table 2). We further analyzed the functions of 78 genes in MSU-treated HEK293 cells through the KEGG pathway and found that amino acid metabolism was affected (Table 1). We also analyzed these genes using Enrichr (37–39). Five of the 26 tools in Enrichr showed that amino acid metabolism was affected (see Supplementary Figure 3; more details at https://maayanlab.cloud/Enrichr/enrich?dataset=4f1656f33465251920dc111be4070988). In addition to gene expression, we analyzed RNA splicing changes with RNA editing and found significant changes in alternative splicing of *CARD8* with RNA editing on the splice site in MSU- or UA-treated THP-1 cells. Alternative splicing of non-coding RNA AC012313.3 with RNA editing on the splice site induced substantial changes in UA-treated HEK293 cells. These findings suggest that RNA editing was affected by MSU and UA treatment in a genome-wide manner.

**TABLE 1:** KEGG pathway analysis of RNA editing genes that indicates changes in expression levels in MSU-treated HEK293 cells.

Term	*P*-value	Genes
hsa00250:Alanine, aspartate and glutamate metabolism	0.002886	IL4I1, GPT2, ASS1
hsa01230:Biosynthesis of amino acids	0.011792	GPT2, ENO3, ASS1
hsa01100:Metabolic pathways	0.013963	IL4I1, GCDH, COX7B, NAGLU, GPT2, ACSL6, ENO3, ASS1
hsa00220:Arginine biosynthesis	0.045566	GPT2, ASS1

### Effect of uric acid and monosodium urate on RNA alternative splicing

**FIGURE 7:**
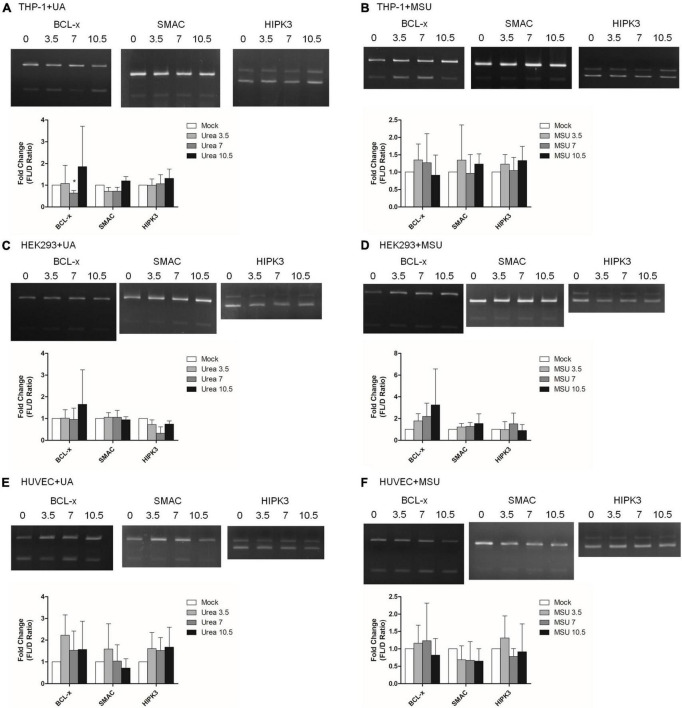
RNA alternative splicing was affected in MSU- or UA-treated cell lines. The alternative splicing changes in *BCL-x*, *SMAC*, and *HIPK3* were detected through RT-PCR in UA-treated THP-1 cells **(A)**, MSU-treated THP-1 cells **(B)**, UA-treated HEK293 cells **(C)**, MSU-treated HEK293 cells **(D)**, UA-treated HUVEC cells €, and MSU-treated HUVEC cells **(F)**. The PCR product sizes of *BCL-x* were 318 and 129 bp, those of *SMAC* were 250 and 118 bp, and those of *HIPK3* were 244 and 181 bp. The bottom bar graphs indicate the fold change of full-length form (FL) and truncated form **(D)** mRNA ratios quantified using LabWorks Image Acquisition and Analysis Software (UVP BioImaging Systems, Upland, CA, United States). Error bars represent standard deviation. Representative results from three independent experiments are presented. The results of three independent experiments were averaged to obtain the final results. *P < 0.05 by Student’s *t*-test, compared with mock cells.

**FIGURE 8:**
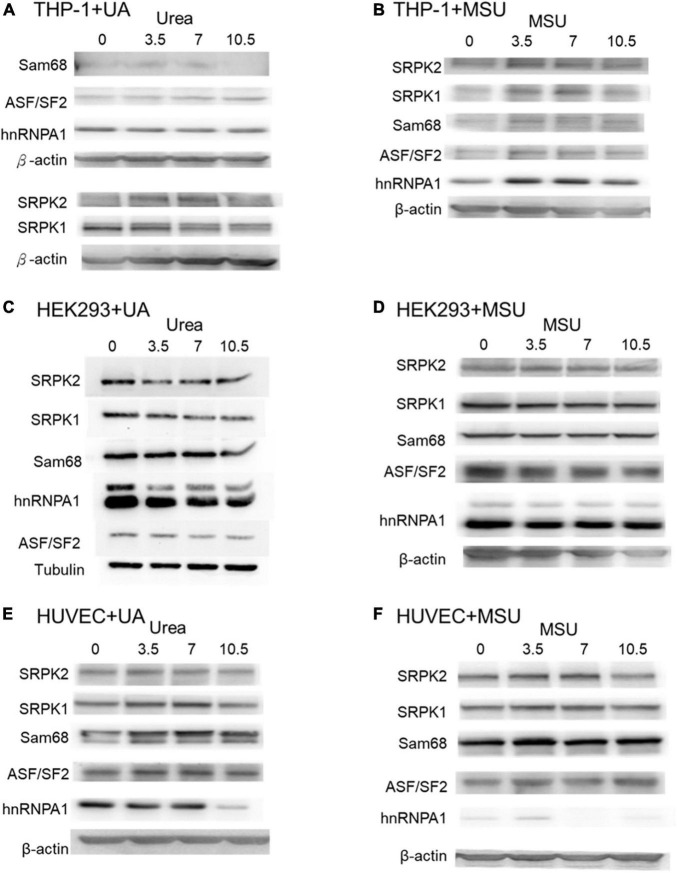
Protein expressions of splicing factors were affected in MSU- or UA-treated cell lines. The expressions of Sam68, ASF/SF2, hnRNPA1, SRPK1, and SRPK2 were detected through Western blotting in UA-treated THP-1 cells **(A)**, MSU-treated THP-1 cells **(B)**, UA-treated HEK293 cells **(C)**, MSU-treated HEK293 cells **(D)**, UA-treated HUVEC cells **(E)**, and MSU-treated HUVEC cells **(F)**. β-actin and tubulin served as the internal control. Representative results from three independent experiments are presented.

In addition to chemical modifications, some other mechanisms are included in epigenomic regulations. For example, mRNA alternative splicing is critical for all cellular processes, as over 90% of all human genes undergo alternative splicing. Many studies have shown that alternative splicing is involved in apoptosis, inflammation, autoimmune diseases, and immune response regulations (40–45), indicating the possibility that alternative splicing is regulated in hyperuricemia and gout. We detected alternative splicing changes of *BCL-x*, *SMAC*, and *HIPK3* in UA- or MSU-treated cell lines; these genes were involved in apoptosis regulation. We found significant changes of *BCL-x* splicing in THP-1 cells treated with UA (Figure 7A). The splicing patterns were also affected in UA- or MSU-treated cell lines, although no significance was observed owing to variation in the patterns (Figures 7B–F). We then detected the expression of splicing factors that had been reported to affect alternative splicing in UA- or MSU-treated cell lines and found that SRPK1 decreased in UA-treated THP-1 cells (Figure 8A). The expression of SRPK1, ASF/SF2, and hnRNPA1 increased in MSU-treated THP-1 (Figure 8B). The expressions of SRPK1 and hnRNPA1 decreased in UA-treated HEK293 cells (Figure 8C), and those of the detected splicing factors did not change significantly in MSU-treated HEK293 cells (Figures 8D,F). The expression of hnRNPA1 decreased and that of Sam68 increased in UA-treated HUVEC cells (Figure 8E). The quantitative results are presented in Supplementary Figure 4. These results indicated that the expressions of splicing factors were affected by UA or MSU treatment and subsequently caused alternative splicing changes. We suspected that the effects of UA or MSU on RNA splicing were universal in the genome. Therefore, we analyzed genome-wide splicing changes by transcriptome sequencing in UA- or MSU-treated THP-1 and HEK293 cells. The ASEs were divided into five groups: SE, MXE, A3SS, A5SS, and RI. We found that 447 splicing events significantly changed in UA-treated THP-1 cells and 431 splicing events changed in MSU-treated THP-1 cells. Similarly, 455 and 435 splicing events significantly changed in UA- and MSU-treated HEK293 cells, respectively (Table 2). These results indicate that alternative splicing was affected by UA or MSU treatment universally in the genome.

**TABLE 2:** Genome-wide analysis of RNA alternative splicing in MSU- or UA-treated cell lines.

AS Types	THP-1 + UA	THP-1 + MSU	HEK293 + UA	HEK293 + MSU
SE	222	222	219	218
RI	69	73	88	93
MXE	47	40	40	26
A5SS	31	42	40	34
A3SS	78	54	68	64

SE, skipped exon; MXE, mutually exclusive exons; A3SS, alternative 3′ splice site; A5SS, alternative 5′ splice site; RI, retained intron.

### RNA modifications, editing, and splicing in patients with gout

**FIGURE 9:**
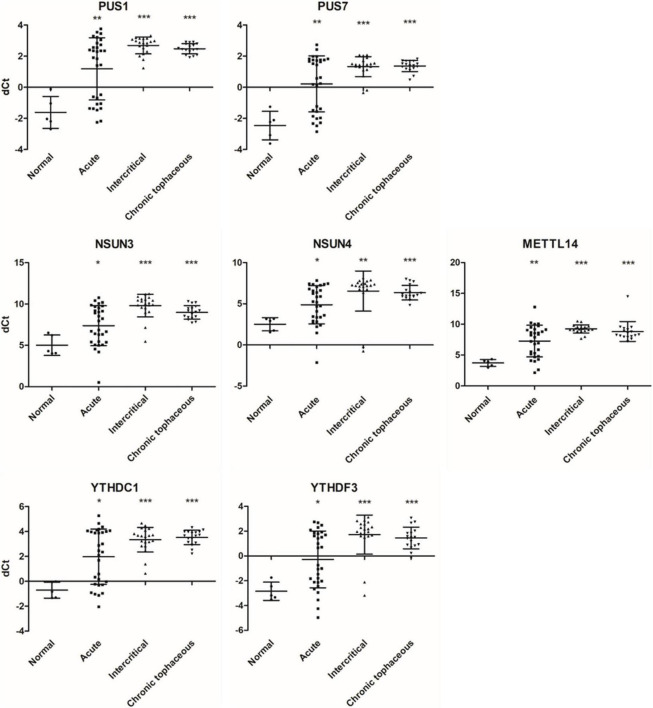
RNA expressions of RNA modification enzymes were affected in patients with gout. The expressions of *PUS1*, *PUS7*, *NSUN3*, *NSUN4*, *METTL14*, *YTHDC1*, and *YTHDF3* were detected through real-time PCR in 67 patients with gout, including 30 patients with acute gout, 20 with intercritical gout, and 17 with chronic tophaceous gout. Five normal samples were used as the control. Lines represent medians, and error bars represent standard deviation. *P < 0.05, **P < 0.005, ***P < 0.0001 by Student’s *t*-test, compared with normal samples. RNA expression levels are shown as dCt (*ALAS1* as internal control).

**FIGURE 10:**
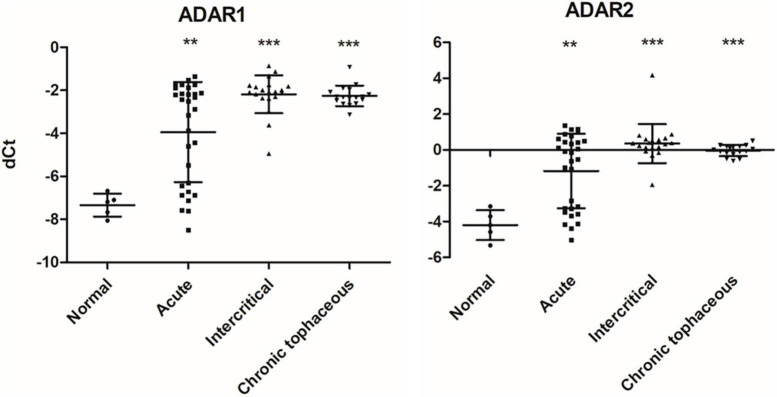
RNA expressions of RNA editing enzymes were affected in patients with gout The expressions of *ADAR1* and *ADAR2* were detected through real-time PCR in 67 patients with gout, including 30 patients with acute gout, 20 with intercritical gout, and 17 with chronic tophaceous gout. Five normal samples were used as the control. Lines represent medians, and error bars represent standard deviation. **P < 0.005, ***P < 0.0001 by Student’s *t*-test, compared with normal samples. RNA expression levels are shown as dCt (*ALAS1* as internal control).

**FIGURE 11:**
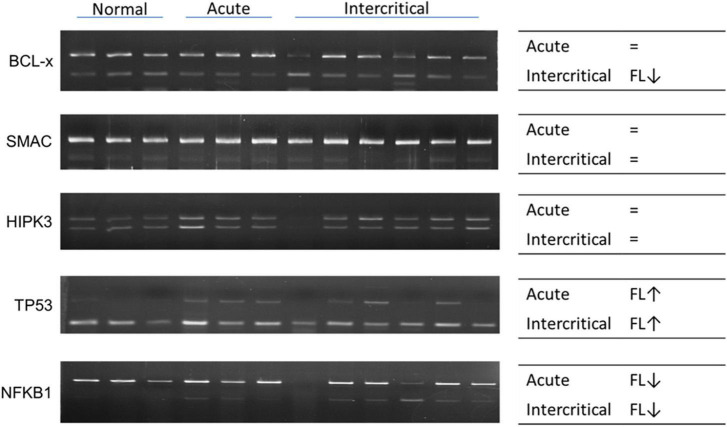
RNA alternative splicing changed in patients with gout. Alternative splicing of *BCL-x*, *SMAC*, *HIPK3*, *TP53*, and *NFKB1* were detected in nine patients with gout, including three patients with acute gout, and six with intercritical gout. Three normal samples were used as the control. Right panels indicate the trend of alternative splicing changes in patients with gout; FL represents full-length; arrow represents increase or decrease; and the sign of equality represents no significant change. The PCR product sizes of *BCL-x* were 318 and 129 bp, those of *SMAC* were 250 and 118 bp, those of *HIPK3* were 244 and 181 bp, those of *TP53* were 201 and 92 bp, and those of *NFKB1* were 346 and 187 bp.

We observed changes in RNA splicing and the expression of RNA modifications and RNA editing enzymes in patients with gout compared with healthy controls. The patients were divided into three groups: acute gout, intercritical gout, and chronic tophaceous gout. The clinical characteristics of patients with gout are presented in Table 3 and Supplementary Figure 5. The expressions of RNA modification enzymes significantly differed between the three patient groups compared with normal controls (Figure 9). The expressions of RNA editing enzymes *ADAR1* and *ADAR2* also decreased significantly in patients with gout (Figure 10). Alternative splicing of *BCL-x* changed in the intercritical gout group. Alternative splicing of *SMAC* and *HIPK3* did not differ significantly between the various patients with gout. We further detected the alternative splicing of *TP53* and *NFKB1* and identified differences in patients with acute and intercritical gout (Figure 11). The quantitative results are presented in Supplementary Figure 6. These results indicate that RNA modifications, editing, and splicing were also affected in gout and may influence the mechanisms of hyperuricemia and gout.

**TABLE 3:** Clinical characteristics of patients with gout.

	Normal	Acute	Intercritical	Chronic Tophaceous
Number of subjects	5	30	20	17
Age (years; mean ± SD)	32.20 ± 3.25	44.87 ± 12.74	56.45 ± 12.36	57.94 ± 18.52
Age range	28∼38	28∼78	39∼87	31∼98
Disease duration (years; mean ± SD)		8.80 ± 7.27	10.90 ± 8.45	18.88 ± 13.31
Disease duration range		0∼23	0∼35	5∼60
Gender				
Male	3	29	20	15
Female	2	1	0	2
BMI (mean ± SD)	23.26 ± 1.73	27.42 ± 4.94	28.36 ± 5.02	28.11 ± 3.82
BMI range	20.2∼25.5	18.4∼38.6	20.38∼39.4	24.1∼38.73
Alcohol consumption				
Yes		9	5	6
No		21	15	11
Uric acid (mg/dl; mean ± SD)		6.99 ± 2.01	5.47 ± 2.14	6.14 ± 1.41
Uric acid range		2.9∼11.4	1.3∼9.8	3.2∼8.7
Serum creatinine (mg/dL; mean ± SD)		1.02 ± 0.30	1.30 ± 0.85	1.09 ± 0.53
Serum creatinine range		0.65∼2.4	0.76∼4.82	0.25∼2.89
Tophus		No	No	Yes
Medications				
NSAID		28	5	2
Colchicine		29	12	10
Benzbromarone		4	15	9
Allopurinol		1	1	3
Feburic		0	3	4
Sulfinpyrazone		0	1	0
Hypertension		3	6	7
Metabolic syndrome		2	5	7

## Discussion

The findings of this study reveal that epigenomic regulations, including RNA modifications, RNA editing, and alternative splicing, are affected in hyperuricemia and gout. We identified different expressions of RNA modification enzymes in different cell lines, indicating tissue-specific regulations of RNA modifications. Accumulating evidence indicates that asymptomatic hyperuricemia is involved in the development of hypertension and chronic kidney disease (46). Hypertension causes activation of the renin–angiotensin system and inhibition of nitric oxide synthesis, which promote endothelial dysfunction and the proliferation of vascular smooth muscle cells. Therefore, HEK293 and HUVEC cell lines were used as cell models in this study. In addition, IL-1 secreted by MSU crystal-stimulated monocytes and macrophages is the starting point of gouty inflammation (47). Hence, the THP-1 cell line was used as one of the cell models in this study. The consistent expressions of NSUN3 at the RNA and protein level in HEK293 cell lines suggest the importance of 5-methylcytosine (m5C) in hyperuricemia and gout. Studies have also reported that N6-methyladenosine (m6A) increases the expression of inflammatory cytokines and inflammatory response (48). RNA modifications may also regulate inflammation and immune response in hyperuricemia and gout. The role and mechanisms of m5C and other RNA modifications warrant further investigation. The inconsistency of mRNA and protein expression may be because of post-translational modifications. Enzyme activity plays a larger role than expression does in the regulation of enzyme functions. RNA modifications can be detected through specific sequencing, such as RIP-seq, Chem-seq, or third-generation sequencing (32). RNA modification changes should be further investigated to elucidate the specific role of RNA modifications in hyperuricemia and gout.

RNA editing has been known to regulate UA metabolism through edited miR-376, which is targeted to phosphoribosyl pyrophosphate synthetase 1, an enzyme involved in the UA synthesis pathway (49). In our study, we determined that RNA editing was also affected in UA- and MSU-treated cells and in patients with gout. The consistent expressions of ADAR1 and ADAR2 at the RNA and protein level in the HEK293 cell line suggest that A-to-I RNA editing is affected by MSU and UA. The results of transcriptome sequencing indicated that RNA editing was affected genome-wide in hyperuricemia and gout. The role and the mechanism of RNA editing in hyperuricemia and gout also warrant further investigation.

RNA alternative splicing is known to be involved in many biological functions. Alternative splicing is affected by many factors, one of them being extracellular pH (50). Acidic extracellular pH also triggers NLRP3 inflammasome activation and affects innate immunity (51). High UA levels in the serum, kidney, and joints of patients with hyperuricemia and gout may cause pH changes in the microenvironment, leading to changes in alternative splicing. Alternative splicing also plays a major role in immune response, indicating that alternative splicing is regulated by UA and MSU and may be involved in the inflammation mechanism in hyperuricemia and gout. Although the number of patients in the sample was low, alternative splicing changes could be observed in patients with gout. To investigate the role and genome-wide changes of alternative splicing, a study involving transcriptome sequencing in patients with hyperuricemia and gout is being planned.

Studies have reported that microRNAs are involved in inflammatory regulation in hyperuricemia and gout. miR-876-5p targets NLRP3 and suppresses MSU-induced inflammation through the TLR4/MyD88/NF-κB pathway in THP-1 macrophages (52). miR-302b can regulate IL-1β production in MSU-induced inflammation by targeting NF-κB and caspase-1 signaling (53). Some miRNAs were observed to be significantly upregulated in the plasma of patients with hyperuricemia and gout. The plasma levels of several miRNA were also observed to correlate with the plasma levels of MCP-1, CRP, serum creatinine, and eGFR (54). The genetic locus rs9952962 of miR-302f identified in a genome-wide association study is associated with the progression of hyperuricemia to gout, and it may affect the inflammation that occurs in gouty arthritis by modulating gene expression (55). In addition, long non-coding RNA (lncRNA), such as ANRIL and AJ227913, were also reported to promote inflammasome activation and to trigger an inflammatory response in gout (56). In this study, we observed RNA editing events in lncRNAs through transcriptome sequencing. The mechanism of RNA editing in lncRNA and the effect of lncRNA on hyperuricemia and gout warrant further investigations. However, studies indicate that epigenetic regulation, such as that by microRNA and lncRNA, plays a key role in hyperuricemia and gout.

Gout is associated with a number of comorbidities, including cardiovascular disease, renal disease, hypertension, diabetes, obesity, and hyperlipidemia (57, 58). Previous studies have demonstrated the role of RNA modifications and RNA editing in hypertension and metabolic syndrome (59–62). Investigations into the cause of hypertension and metabolic syndrome in patients with gout and the role of epigenomic regulations are of critical importance. Only 34.3% patients exhibited hypertension or metabolic syndrome in our gout patient cohort. The recruitment of more patients is necessary to explore the relationship between gout and hypertension or metabolic syndrome.

In this study, we investigated epigenomic regulations in UA- and MSU-treated cell lines and found genome-wide changes in RNA editing and alternative splicing. However, the epigenomic regulations in patients with hyperuricemia and gout warrant further investigation. We plan to conduct future studies using transcriptome sequencing to study the role of epigenomic regulations in such patients.

## Data availability statement

Original datasets are available in a publicly accessible repository: NCBI Sequence Read Archive (SRA). The original contributions presented in the study are publicly available. This data can be found here: PRJNA870964 (https://dataview.ncbi.nlm.nih.gov/object/PRJNA870964).

## Ethics statement

The studies involving human participants were reviewed and approved by China Medical University and Hospital Research Ethics Committee. The patients/participants provided their written informed consent to participate in this study.

## Author contributions

C-MH, P-HH, and J-GC designed the study. Y-CC, I-LL, Y-TL, J-CY, C-LL, and T-YL carried out the experiments. H-DC and S-JT performed the bioinformatic analyses. Y-CC and T-YL wrote the manuscript in consultation with C-MH, P-HH, and J-GC. J-GC supervised the project. All authors contributed to the article and approved the submitted version.
