# Supplementary material for: Exploring RNA modifications, editing, and splicing changes in hyperuricemia and gout
Source: Front Med (Lausanne). 2022 Sep 6;9:889464. doi: 10.3389/fmed.2022.889464 (PMC9487523; doi:10.3389/fmed.2022.889464)
Supplement: Supplementary file 1 [file Data_Sheet_1.docx]

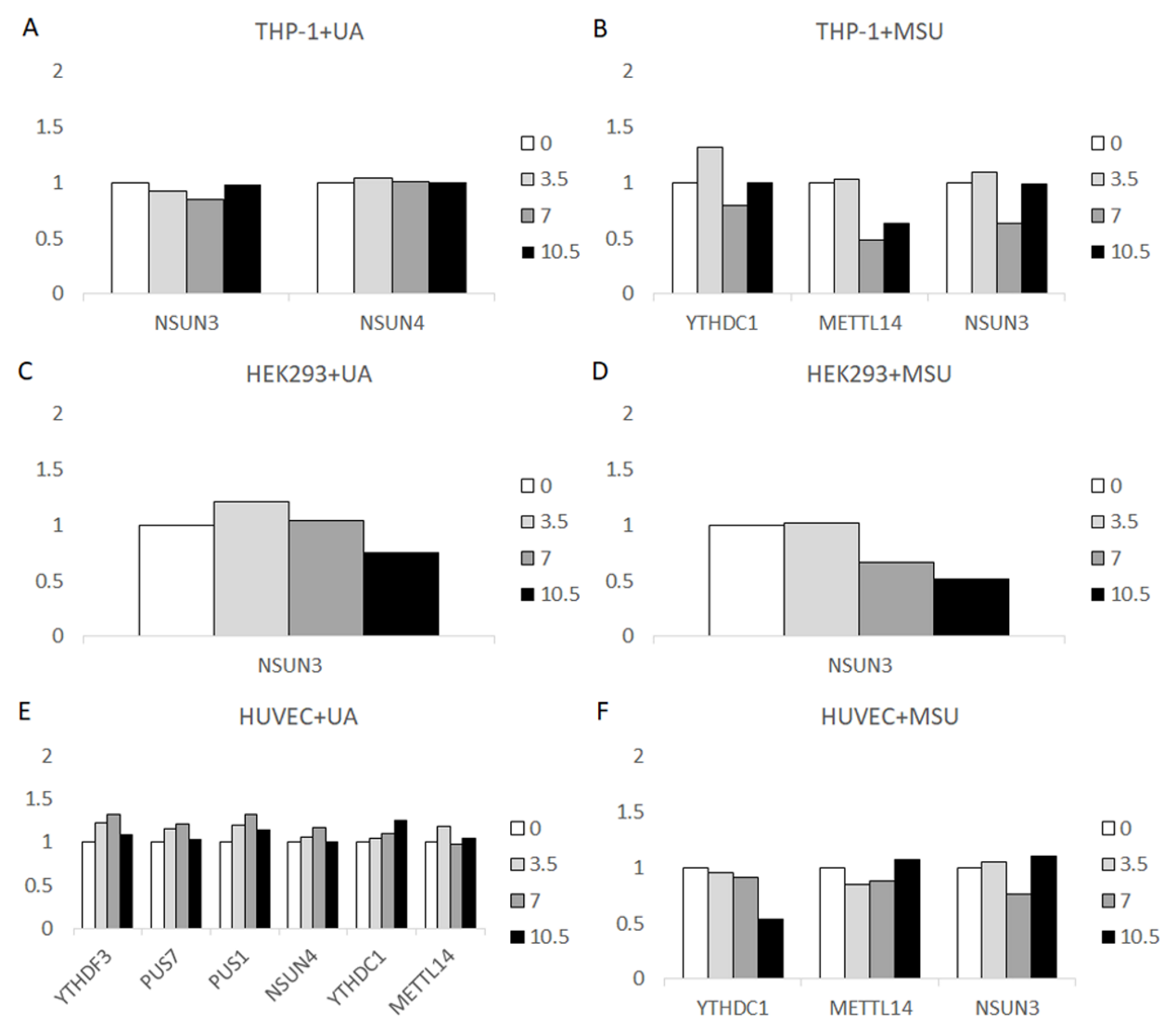


Supplementary Figure 1. Quantitative results of protein expression of RNA modification enzymes in MSU- or UA-treated cell lines.

Changes in expression of RNA modification enzymes with RNA levels were detected through Western blot. (A) NSUN3 and NSUN4 were detected in UA-treated THP-1 cells. (B) YTHDC1, METTL14, and NSUN3 were detected in MSU-treated THP-1 cells. (C, D) NSUN3 was detected in UA-and MSU-treated HEK293 cells. (E) YTHDF3, PUS7, PUS1, NSUN4, YTHDC1, and METTL14 were detected in UA-treated HUVEC cells. (F) YTHDC1, METTL14, and NSUN3 were detected in MSU-treated HUVEC cells.


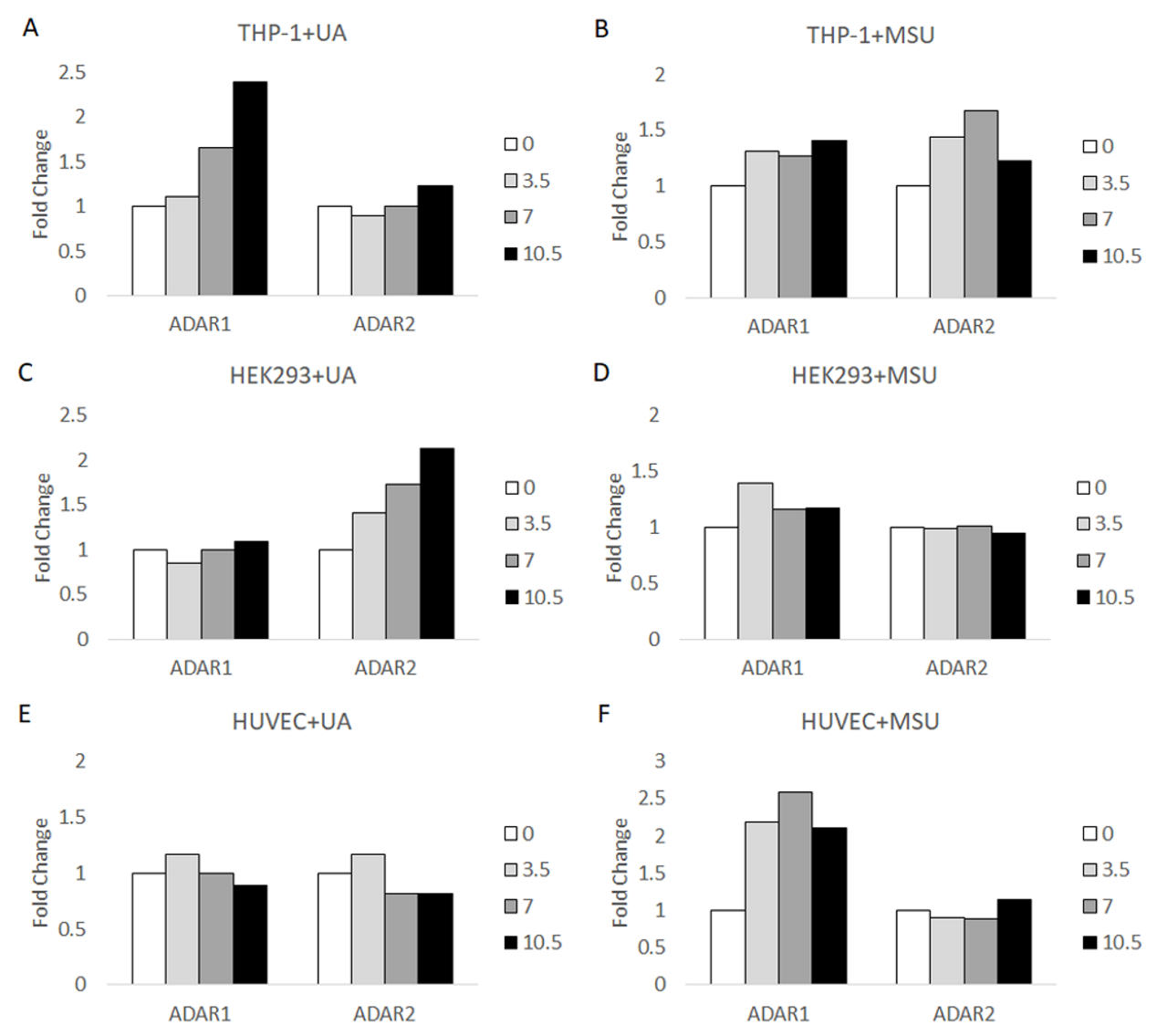


Supplementary Figure 2. Quantitative results of protein expression of RNA-editing enzymes in MSU- or UA-treated cell lines.

Expression of ADAR1 and ADAR2 was detected through Western blot in UA-treated THP-1 cells (A), MSU-treated THP-1 cells (B), UA-treated HEK293 cells (C), MSU-treated HEK293 cells (D), UA-treated HUVEC cells (E), and MSU-treated HUVEC cells (F).


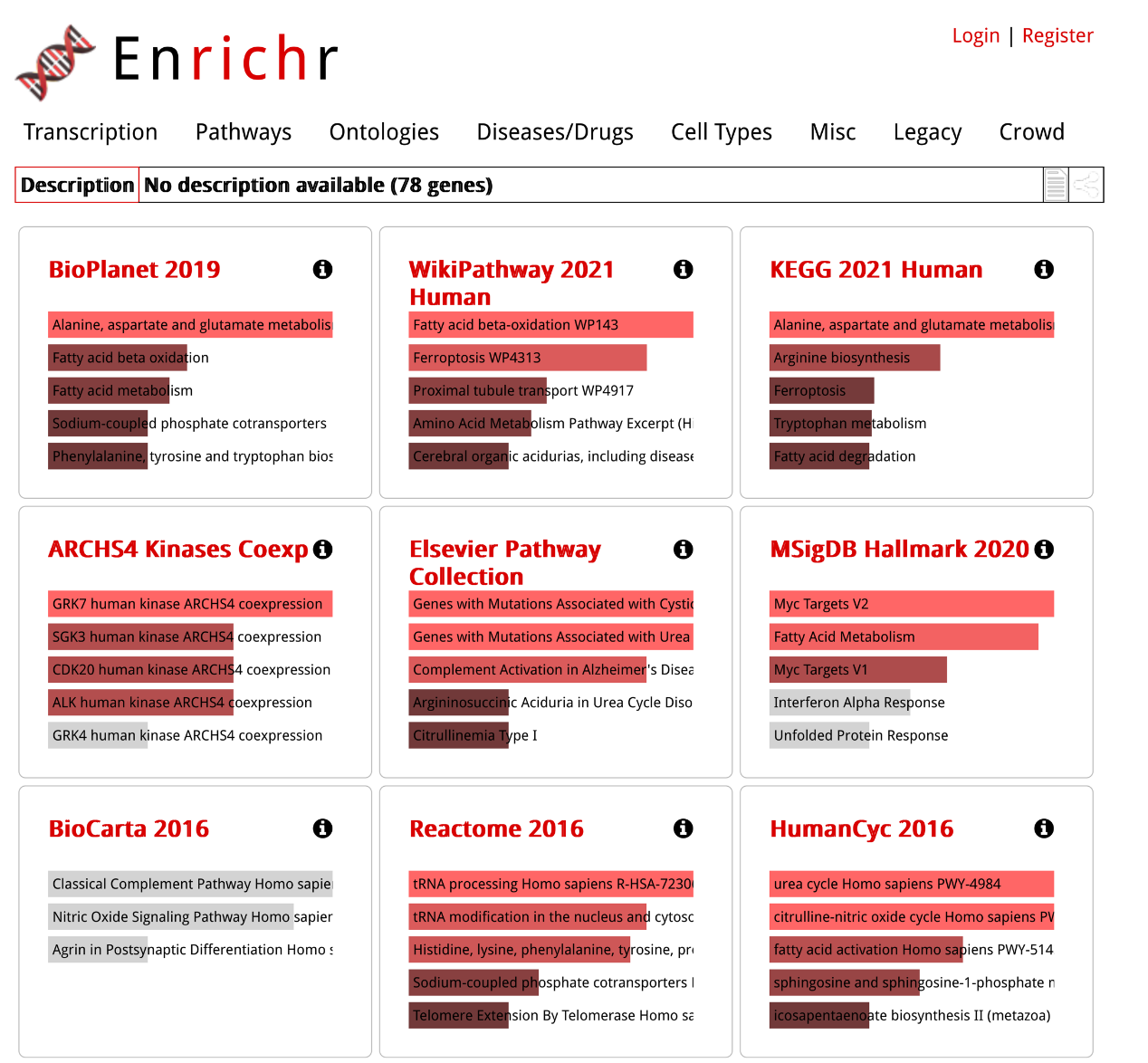


Supplementary Figure 3. Enrichr analysis of RNA-editing genes indicating changes in expression levels in MSU-treated HEK293 cells.

Enrichr contains 26 tools for analysis, and 5 of these demonstrated that amino acid metabolism was affected. The analysis results are partially presented. More details at https://maayanlab.cloud/Enrichr/enrich?dataset=4f1656f33465251920dc111be4070988https://maayanlab.cloud/Enrichr/enrich?dataset=4f1656f33465251920dc111be4070988


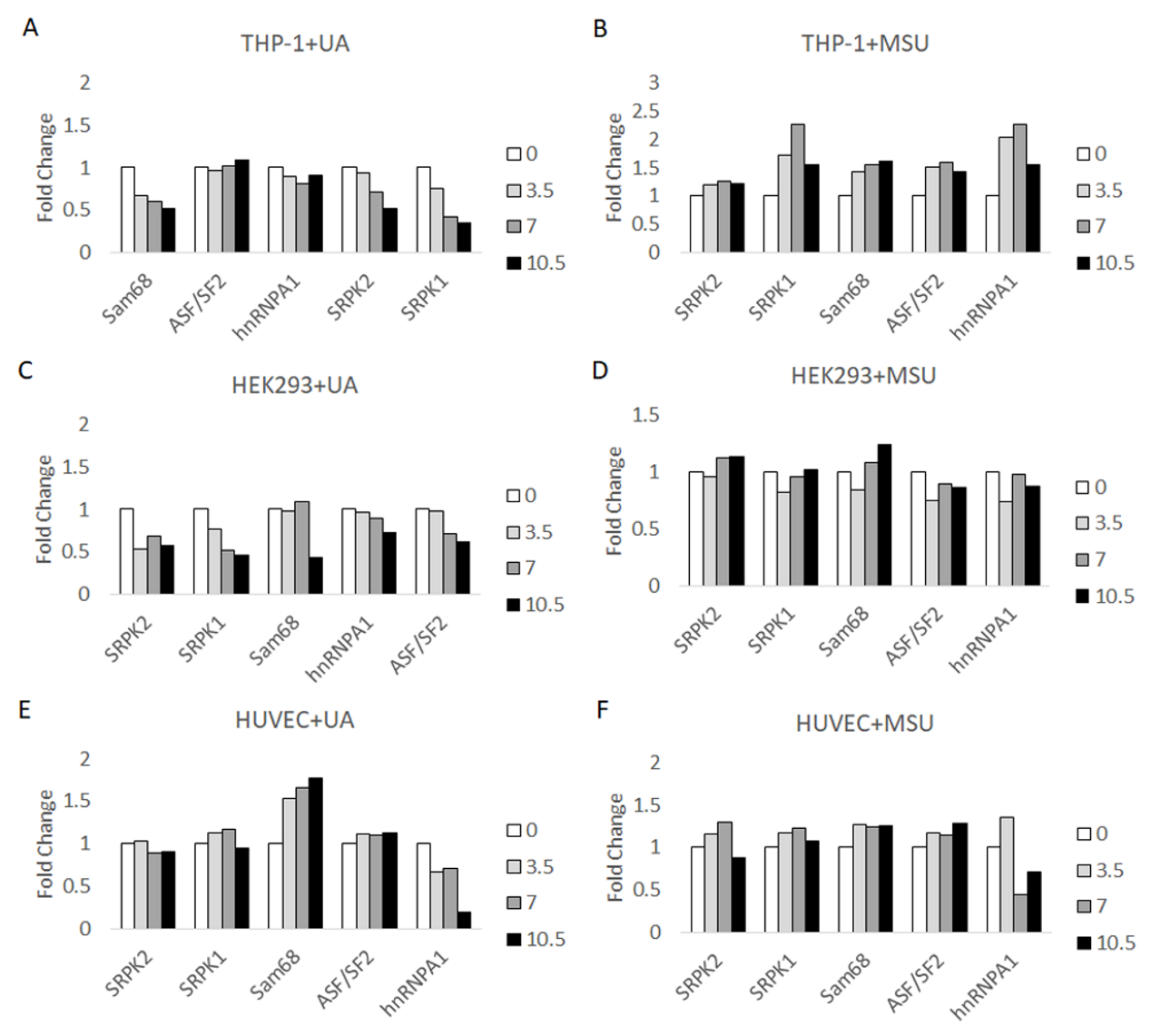


Supplementary Figure 4. Quantitative results of protein expression of splicing factors in MSU- or UA-treated cell lines.

Expression of Sam68, ASF/SF2, hnRNPA1, SRPK1, and SRPK2 was detected through Western blot in UA-treated THP-1 cells (A), MSU-treated THP-1 cells (B), UA-treated HEK293 cells (C), MSU-treated HEK293 cells (D), UA-treated HUVEC cells (E), and MSU-treated HUVEC cells (F).


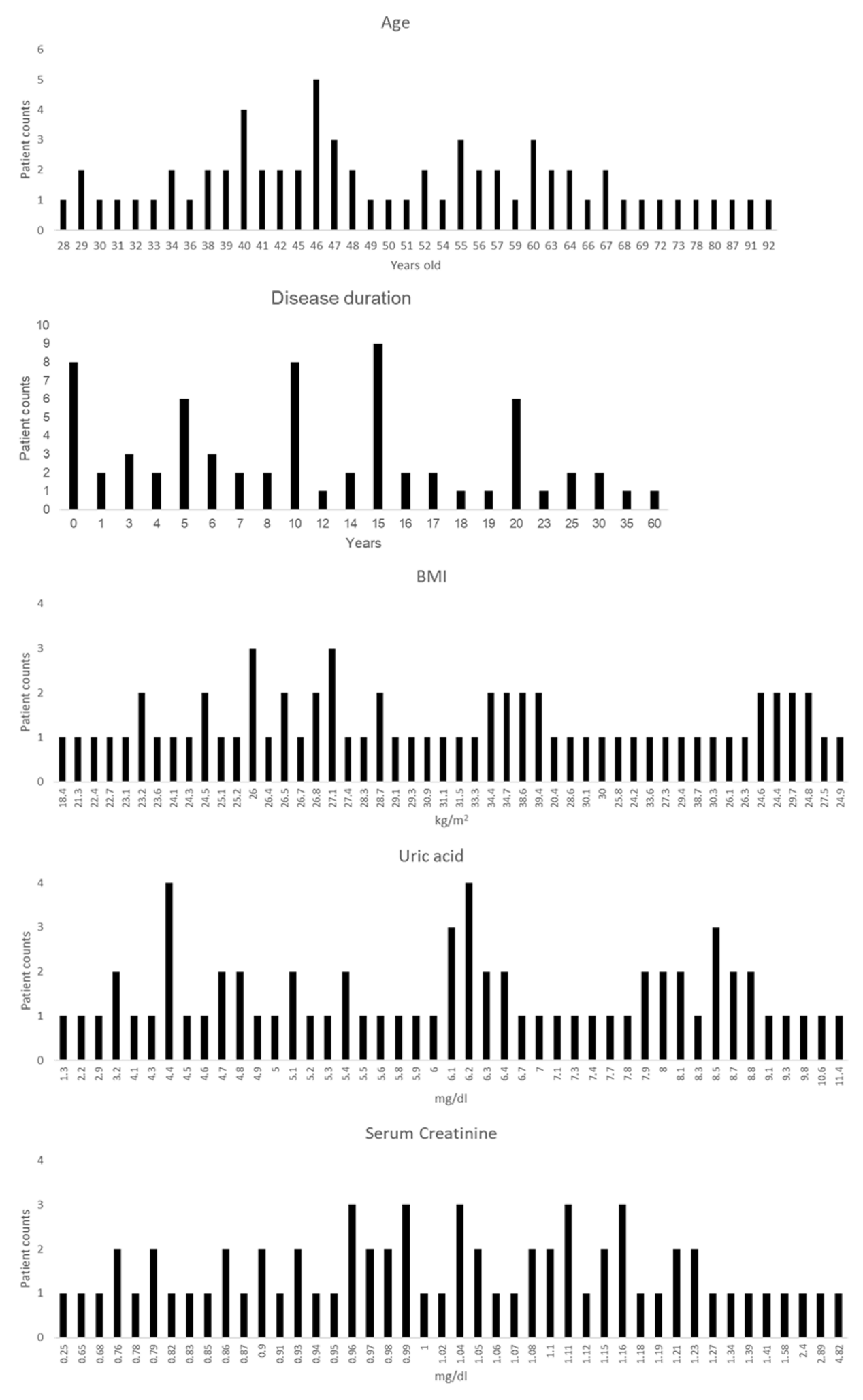


Supplementary Figure 5. Histogram displaying clinical characteristics of patients.

Histograms showing patient counts for the variables of age, disease duration, BMI, uric acid, and serum creatinine.


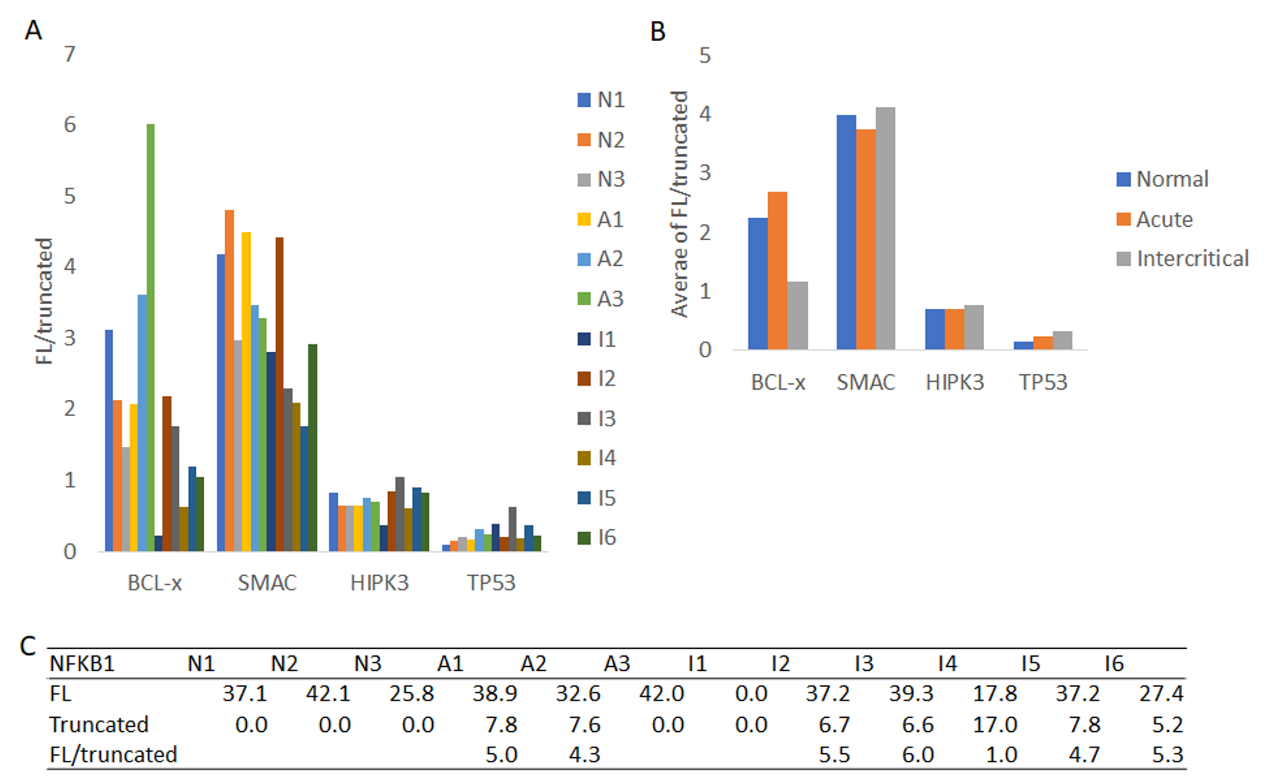


Supplementary Figure 6. Quantitative results of RNA alternative splicing in patients with gout.

Alternative splicing of *BCL-x*, *SMAC*, *HIPK3*, *TP53*, and *NFKB1* was detected in nine patients with gout, including three patients with acute gout and six with intercritical gout. Three healthy samples were used as the control. The full-length form (FL) and truncated form were quantified and the ratio calculated. (A) The ratio of *BCL-x*, *SMAC*, *HIPK3*, *TP53* are presented for each patient. N1, N2, and N3 represent healthy samples. A1, A2, and A3 represent patients with acute gout. I1, I2, I3, I4, I5, and I6 represent patients with intercritical gout. (B) Average ratio of *BCL-x*, *SMAC*, *HIPK3*, *TP53* in each group. (C) Quantitative results of NFKB1 of each sample are presented. The ratio of some samples is not presented because the truncated form was too weak to quantify.

Supplementary Table S1. Primers used in the study

| Genes | Forward | Reverse |
| --- | --- | --- |
| PUS1 | gtgtggctgattgacgacat | cacatctgttcttggagttaaacc |
| PUS3 | tgtaagtttgaaaatgtcaagtgga | ccacagttgttgtaggtgggta |
| RPUSD2 | gagagaattcacgagcaggttc | ttacaggtcacttcctcagtgg |
| PUS7 | catggactcactgaggctga | ccgagaacccttgatgagaa |
| NSUN2 | gaggtggacggtcagaaagt | atttgtgtgccaggcaagtt |
| NSUN3 | gctcctggagggaaatcaat | ctgccttagccacctcaatc |
| NSUN4 | taattctggccattcctgct | gcagcctcctaagccacat |
| NSUN5 | gaaacgcagcgctactcc | ggccgcagcttcttctcc |
| NSUN6 | cagtgtactgccccgttactt | caggtgctgcacacaagtct |
| hNOP2 | agctgctcggaagcagaag | cggtctcctcttcactccac |
| TRDMT1 | ccctctagacccttgccaat | tcagttcccccgtctccta |
| ALKBH5 | ggcctcatcctcatgtcatc | tgagctctggccttggat |
| FTO | ttttctactcagagggagaatagctc | cgcctcctgcatgtagttct |
| METTL14 | gccttcatctatttggaagagatagt | ttgctatttgtaagcgttggtc |
| METTL3 | caggctcaacatacccgtact | acattctctccccaactcca |
| YTHDC1 | taaacagcgcagggcatt | gagggaagggaaacagatgg |
| YTHDF2 | gaaaggccccgtttacttactt | atttctgccacgccacag |
| YTHDF3 | tgtggactataatgcgtatgctg | ttcaaatttgcccttccact |
| ADAR1 | ttcgagaatcccaaacaagg | ctggattccacagggattgt |
| ADAR2 | ctcacgctcgcagaaaagt | tcaccttggcatctttaacatc |
| BCL-x | gacgagtttgaactgcggta | tgctgcattgttcccataga |
| SMAC | agtaaccctgtgtgcggttc | ctgccacacttcatcttcctc |
| HIPK3 | agcctgccactaccaagaaa | cagcaatttcttgcctctcc |
| TP53 | ccagccaaagaagaaaccac | cctcattcagctctcggaac |
| NFKB1 | tggaagcacgaatgacagag | atttcctcccctccagtcac |

Supplementary Table S2. RNA-editing variants correlated with gene expression levels

| MSU-treated THP-1 | UA-treated THP-1 | MSU-treated HEK293 | UA-treated HEK293 |
| --- | --- | --- | --- |
| GATD3B | FADS2 | ABHD11 | ADAT3 |
| GLO1 | IL21R | AC004253.1 | AJUBA |
| IL21R | SIGLEC11 | AC007566.1 | METTL7A |
| SIGLEC6 | SIGLEC6 | AC008267.3 | SPSB3 |
|  |  | AC011462.1 |  |
|  |  | AC020928.1 |  |
|  |  | AC021087.5 |  |
|  |  | AC027559.1 |  |
|  |  | AC087741.1 |  |
|  |  | AC096677.1 |  |
|  |  | AC138904.1 |  |
|  |  | AC138904.3 |  |
|  |  | ACSL6 |  |
|  |  | AL162595.1 |  |
|  |  | AL355388.2 |  |
|  |  | AP000347.1 |  |
|  |  | AP001469.3 |  |
|  |  | ARHGEF6 |  |
|  |  | ASPHD1 |  |
|  |  | ASS1 |  |
|  |  | ATPSCKMT |  |
|  |  | C10orf143 |  |
|  |  | C1S |  |
|  |  | CAMLG |  |
|  |  | CARD14 |  |
|  |  | CCDC18-AS1 |  |
|  |  | CEACAM19 |  |
|  |  | COX7B |  |
|  |  | DNAJC22 |  |
|  |  | EEF2KMT |  |
|  |  | ENO3 |  |
|  |  | FAM221A |  |
|  |  | FAM227A |  |
|  |  | GABPB1-AS1 |  |
|  |  | GCDH |  |
|  |  | GPT2 |  |
|  |  | GRIN2D |  |
|  |  | GSDMB |  |
|  |  | IFRD1 |  |
|  |  | IL4I1 |  |
|  |  | IQCH |  |
|  |  | JPT1 |  |
|  |  | KCNG1 |  |
|  |  | LINC00963 |  |
|  |  | LTBP4 |  |
|  |  | MIR34AHG |  |
|  |  | MRTO4 |  |
|  |  | NAGLU |  |
|  |  | NBR2 |  |
|  |  | NEBL |  |
|  |  | NHP2 |  |
|  |  | OVOL2 |  |
|  |  | PGBD4 |  |
|  |  | PILRA |  |
|  |  | POLN |  |
|  |  | PUS1 |  |
|  |  | RAB39A |  |
|  |  | RBM3 |  |
|  |  | SBK1 |  |
|  |  | SFXN2 |  |
|  |  | SGPP2 |  |
|  |  | SLC25A29 |  |
|  |  | SLC34A3 |  |
|  |  | SLC3A2 |  |
|  |  | TEX19 |  |
|  |  | TMC7 |  |
|  |  | TMEM9B-AS1 |  |
|  |  | TRAFD1 |  |
|  |  | TRMT9B |  |
|  |  | TSHZ3 |  |
|  |  | UGDH-AS1 |  |
|  |  | WDR31 |  |
|  |  | WDR4 |  |
|  |  | WDR78 |  |
|  |  | XPOT |  |
|  |  | ZNF581 |  |
|  |  | ZNF829 |  |
|  |  | ZSCAN30 |  |
